# Effect of Behavior Modification on Outcome in Early- to Moderate-Stage Chronic Kidney Disease: A Cluster-Randomized Trial

**DOI:** 10.1371/journal.pone.0151422

**Published:** 2016-03-21

**Authors:** Kunihiro Yamagata, Hirofumi Makino, Kunitoshi Iseki, Sadayoshi Ito, Kenjiro Kimura, Eiji Kusano, Takanori Shibata, Kimio Tomita, Ichiei Narita, Tomoya Nishino, Yoshihide Fujigaki, Tetsuya Mitarai, Tsuyoshi Watanabe, Takashi Wada, Teiji Nakamura, Seiichi Matsuo, Study Group for Frontier of Renal Outcome Modifications in Japan (FROM-J)

**Affiliations:** 1 Department of Nephrology, Faculty of Medicine, University of Tsukuba, Tsukuba, Ibaraki, Japan; 2 Department of Medicine and Clinical Science, Okayama University Graduate School of Medicine, Dentistry, and Pharmaceutical Sciences, Okayama, Japan; 3 Dialysis Unit, University Hospital of the Ryukyus, Okinawa, Japan; 4 Division of Nephrology, Endocrinology, and Vascular Medicine, Tohoku University Graduate School of Medicine, Sendai, Japan; 5 Department of Nephrology and Hypertension, St. Marianna University School of Medicine, Kanagawa, Japan; 6 Division of Nephrology, Department of Medicine, Jichi Medical University, Tochigi, Japan; 7 Division of Nephrology, Department of Medicine, Showa University School of Medicine, Tokyo, Japan; 8 Department of Nephrology, University of Kumamoto, Kumamoto, Japan; 9 Division of Clinical Nephrology and Rheumatology, Niigata University Graduate School of Medical and Dental Science, Niigata, Japan; 10 Division of Nephrology, Second Department of Internal Medicine, Nagasaki University School of Medicine, Nagasaki, Japan; 11 Internal Medicine 1, Hamamatsu University School of Medicine, Shizuoka, Japan; 12 Division of Nephrology and Hypertension, Saitama Medical Center, Saitama Medical School, Saitama, Japan; 13 Department of Internal Medicine III, Fukushima Medical University School of Medicine, Fukushima, Japan; 14 Division of Nephrology, Department of Laboratory Medicine, Institute of Medical, Pharmaceutical, and Health Sciences, Faculty of Medicine, Kanazawa University, Ishikawa, Japan; 15 Japan Dietitian Association, Tokyo, Japan; 16 Department of Nephrology, University of Nagoya, Aichi, Japan; University of Perugia, ITALY

## Abstract

**Objectives:**

Owing to recent changes in our understanding of the underlying cause of chronic kidney disease (CKD), the importance of lifestyle modification for preventing the progression of kidney dysfunction and complications has become obvious. In addition, effective cooperation between general physicians (GPs) and nephrologists is essential to ensure a better care system for CKD treatment. In this cluster-randomized study, we studied the effect of behavior modification on the outcome of early- to moderate-stage CKD.

**Design:**

Stratified open cluster-randomized trial.

**Setting:**

A total of 489 GPs belonging to 49 local medical associations (clusters) in Japan.

**Participants:**

A total of 2,379 patients (1,195 in group A (standard intervention) and 1,184 in group B (advanced intervention)) aged between 40 and 74 years, who had CKD and were under consultation with GPs.

**Intervention:**

All patients were managed in accordance with the current CKD guidelines. The group B clusters received three additional interventions: patients received both educational intervention for lifestyle modification and a CKD status letter, attempting to prevent their withdrawal from treatment, and the group B GPs received data sheets to facilitate reducing the gap between target and practice.

**Main outcome measure:**

The primary outcome measures were 1) the non-adherence rate of accepting continuous medical follow-up of the patients, 2) the collaboration rate between GPs and nephrologists, and 3) the progression of CKD.

**Results:**

The rate of discontinuous clinical visits was significantly lower in group B (16.2% in group A vs. 11.5% in group B, p = 0.01). Significantly higher referral and co-treatment rates were observed in group B (p<0.01). The average eGFR deterioration rate tended to be lower in group B (group A: 2.6±5.8 ml/min/1.73 m^2^/year, group B: 2.4±5.1 ml/min/1.73 m^2^/year, p = 0.07). A significant difference in eGFR deterioration rate was observed in subjects with Stage 3 CKD (group A: 2.4±5.9 ml/min/1.73 m^2^/year, group B: 1.9±4.4 ml/min/1.73 m^2^/year, p = 0.03).

**Conclusion:**

Our care system achieved behavior modification of CKD patients, namely, significantly lower discontinuous clinical visits, and behavior modification of both GPs and nephrologists, namely significantly higher referral and co-treatment rates, resulting in the retardation of CKD progression, especially in patients with proteinuric Stage 3 CKD.

**Trial registration:**

The University Hospital Medical Information Network clinical trials registry UMIN000001159

## Introduction

It is estimated that more than 10% of the general population has chronic kidney disease (CKD) in Japan [1] and other countries, increasing with the aging of the population [2]. Previous studies have suggested that CKD is not only main risk of end-stage kidney disease (ESKD), but one of the most important risk factors for cardiovascular disease among known risk factors of diabetes, hypertension, hyperlipidemia, obesity, smoking, and lifestyle-related diseases [3]^,^[4]. Therefore, early detection and early treatment start are also important in terms of preventing increases in the ESKD population and cardiovascular complications [2]. However, even if early detection of CKD is achieved, effective treatment and care systems are not yet established. At the onset of lifestyle-related diseases such as diabetes, hypertension, dyslipidemia, and CKD, most subjects consult general practitioners (GPs). Furthermore, there is initially a lack of subjective symptoms, until severe organ failure occurs. Owing to the asymptomatic phases of CKD, patients are left untreated until ESKD or advanced symptomatic phases. In a previous study, many subjects did not accept medical follow-up or stopped attending the follow-up even when it was necessary, especially younger and male patients [5]. Consequently, it is important to establish appropriate, consistent, and specific treatment and prevention-based care systems according to the progression of CKD.

In this study, we performed a prospective stratified cluster-randomized trial in order to examine the effectiveness of behavior changes of both patients and GPs, designed to prevent non-adherence of continuous medical follow-up in CKD patients, with appropriate timing of introductions from GPs to nephrologists and co-treatment by GPs and nephrologists in order to reduce the evidence-practice gap in CKD treatment, and with the establishment of a better outcome of CKD patients seen by GPs at local medical associations (LMA) by adding several advanced care systems for CKD patients. Previously, several follow-up studies of CKD patients at annual screening were performed in the general population [6] or at nephrology clinics [7], but no long-term follow-up studies or clinical studies of CKD patients cared for by GPs have been performed. Furthermore, there is little information about the detailed clinical outcomes and treatment patterns at GPs. We compared the effects of lifestyle modification by educational intervention on mainly early-stage CKD patients seen at GPs.

In this study, we show the effect of an advanced CKD care system on early-stage CKD patients seen at GPs, and elucidate their outcomes. A cluster-randomized design that randomized LMA was chosen to avoid within-LMA contamination.

## Methods

### Study design

The study methods have been reported previously [8]. The detailed study design was developed and implemented by an advisory committee, and all analyses were completed by a coordinating center (for details, see supporting information). This study was a Central Institutional Review Board Program; the Committee on Ethics in Strategic Research of the Kidney Foundation, Japan, and the institutional review board at the University of Tsukuba examined and approved the implementation plans and their revision.

This study was conducted at 15 managing facilities in Japan. Two to four local medical associations (LMAs), which constitute the infrastructure of the Japan Medical Association, located near each managing facility, were selected. In total, 49 LMAs (for details, see supporting information and S1 Fig) participated. The trial was a stratified open cluster-randomized study with two intervention groups of A (standard intervention) and B (advanced intervention). Each LMA was regarded as a cluster. For randomization, we divided the country into four regions as strata because the rate of increase of dialysis patients varies from region to region in Japan [9]. Each LMA recruited 10~58 GPs by whom patients in this study were treated.

The study was sponsored by a grant for a strategic outcome study project from the Ministry of Health, Labour and Welfare of Japan.

### Study patients

Each registered GP obtained written informed consent for the study from eligible patients before randomization. Eligible participants were those aged between 40 and 74 years who had Stage 1, 2 4, or 5 CKD, or Stage 3 CKD with proteinuria (ratio of urinary protein/urinary creatinine ≥ 0.3 g/g.cre, or proteinuria ≥ 1+) and diabetes or Stage 3 CKD with proteinuria (ratio of urinary protein/urinary creatinine >0.3 g/g.cre or proteinuria >1+) and hypertension, who were under consultation with GPs. Dialysis patients, renal transplant patients, and those who did not consent were excluded from the study.

**Fig 1:**
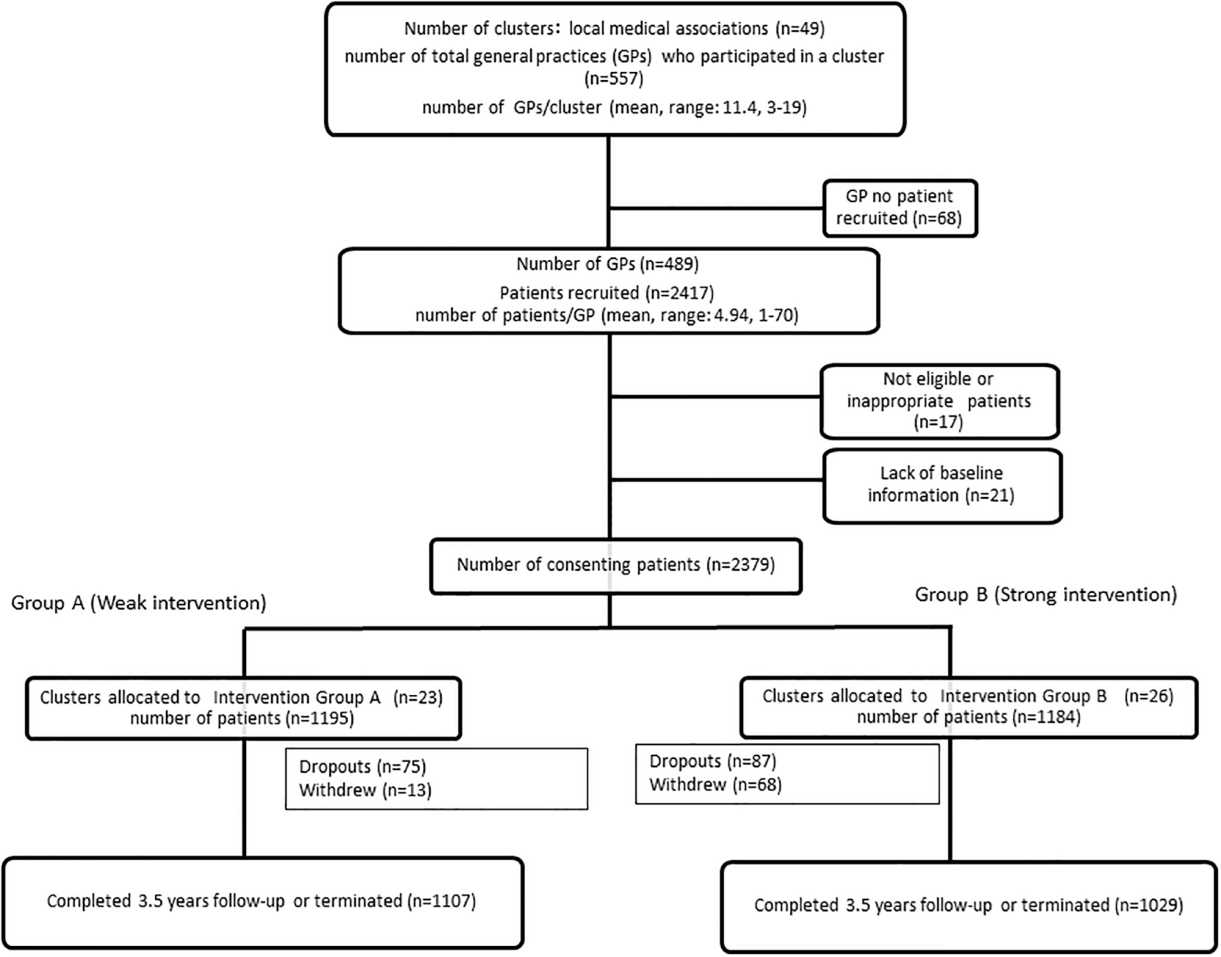
Study clusters and patients. We recruited 49 local medical associations (clusters) in 15 different prefectures, which were classified into four regions (strata) based on the level of increase in the rate of dialysis patients [9]. We recruited 557 GPs and 2,417 patients; 2,379 patients from 489 GPs gave consent. After randomization, 68 patients in group B chose to withdraw, while only 13 patients in group A did so. Most of the patients in group B withdrew just after randomization due to an aversion to the educational intervention. Finally, 1,107 patients in group A and 1,029 patients in group B completed this cluster-randomized trial.

Participant recruitment was from April 1, 2008 to October 19, 2008. On October 20, 2008, the LMAs were randomly assigned at a ratio of 1:1 to group A (standard intervention) or group B (advanced intervention) (Fig 1). Randomization was performed centrally by means of a computer-generated random-number sequence. The primary intervention study and follow-up duration lasted from October 20, 2008, to March 31, 2012.

### Study assessments

At each consultation, physicians and their certified staff measured patients’ blood pressure, and checked their blood pressure readings recorded at home. Examinations or surveys were performed every six months regarding body weight, smoking status, obtained blood and urine, number of patients referred by nephrologists, number of new dialysis patients, and incidence of cardiovascular events. In group B, physicians sent a list of patients who did not visit their clinics as scheduled to the coordinating center every month.

The standards for referral from GPs to nephrologists were as follows: 1) ratio of urinary protein/urinary creatinine ≥ 0.5 or proteinuria ≥ 2+; 2) both proteinuria and hematuria are positive (≥ 1+); 3) estimated GFR (eGFR) < 50 mL/min/1.73 m^2^; and 4) when GPs judge that patients should consult a nephrologist.

eGFRs in this study were calculated using the following formula:

$$\text{eGFR }\left(\text{mL/min/1}{\text{.73 m}}^{\text{2}}\right){\text{=194 x Age}}^{\text{-0}.287}{\text{x Cre}}^{\text{-1}.094}\left(\text{x 0}.739\text{ in the case of women}\right)$$

### Study interventions

Patients in both group A and group B clusters underwent treatment in accordance with the current CKD treatment guide. S1 Table shows a summary of targets for CKD treatment applied to all patients. In patients with CKD, lifestyle modifications to avoid obesity and stop smoking are necessary. In addition, strict blood pressure control (less than 130/80 mmHg), strict blood sugar control (Hba1c <6.9%), and non-HDL cholesterol control (non-HDL-C <150 mg/dl) are targets for CKD treatment.

Lecture meetings about the CKD treatment guidelines were held several times annually at local medical associations, and the coordinating center sent a newsletter about CKD treatment to general practitioners bimonthly.

Patients in the group B clusters received three additional interventions. Firstly, the group B patients received 30-minute educational sessions from dieticians upon visiting their local GP offices every three months. The curricula by the dieticians were developed centrally. In terms of lifestyle and nutritional modification, patients received a 30-minute session from a registered dietician. Prior to every session, the dietician checked the patient’s condition using a checklist as shown in S2 Fig. Every checklist item had a score; the higher the score, the further the treatment target. The dietician decided on the dietary counseling theme depending on the score. Registered dieticians provided lifestyle/dietary advice according to the instructions. They helped patients to achieve their CKD treatment goals (S1 Table), explained the examination results, achievements in CKD care, and their implications to patients. The registered dieticians had received training so that they would be able to provide integrated and consistent advice. Secondly, the group B patients received a CKD treatment report from the coordinating center bimonthly to learn about CKD and their ideal lifestyle to prevent progression. In addition, the patients also received a letter, phone call, or email a week before the consultation day, and those who had not consulted a physician for over two months were encouraged to receive care, as an attempt to prevent their withdrawal from treatment. Thirdly, the group B GPs received comments about their patients’ data, focusing on the gap between target and practice, from the coordinating center. The coordinating center also provided information on the patients scheduled to visit the office, examinations, treatment patients should undergo on their next visitation, patients who did not visit hospitals as scheduled, those who were going to receive lifestyle/dietary advice, and those who met the conditions for referral to nephrologists.

### Study outcomes

There were three primary outcomes in this study. The first primary parameter for assessment was the rate of discontinuation of clinical visits. The definition of discontinuation of clinical visits was the absence of a clinical visit for more than 6 months. We compared the rate of discontinuous clinical visits between the groups. The second primary parameter was the proportion of patients under co-treatment between GPs and nephrologists. For calculating the proportion of patients under co-treatment between GPs and nephrologists, we counted the number of patients who reached the criteria for requiring nephrology care, and the number of patients who received an introductory letter to a nephrologist using the standards for referral from GPs to nephrologists. Furthermore, we counted the number of return visits to GPs after referral to nephrologists. The third primary parameter for assessment was the progression of CKD stage. To compare annual changes in the CKD stage between the two groups, we used annual eGFR changes (ml/minute/1.73 m^2^/year) in total and in each CKD stage.

Secondary parameters were as follows: 1) the proportion of adherence to the complete CKD treatment guide, 2) the rate of achievement of blood pressure goals, 3) the number of subjects with a 50% reduction in urinary protein, 4) the number of subjects with a doubling of serum creatinine or a 50% reduction in eGFR, 5) yearly changes in the number of patients starting renal replacement therapy, and 6) the incidence of cardiovascular events.

### Sample size calculation

To determine the sample size, we compared the rate of exacerbation of eGFR between group A and group B interventions. We referred to the epidemiological data in the CKD taskforce, collected by the statistical board of the Japanese Society of Nephrology, in which the worsening velocity was -0.59 (SD 0.04) ml/min/year based on changes in renal function among healthy Japanese people who underwent health checkups [10], and the rate of renal deterioration in patients in Stage 3 CKD with diabetes or hypertension [mean serum creatinine = 1.69 mg/dl (SD = 0.57 mg/dl), annual decrease rate = 5.93 ml/min/year (SD 4.321 ml/min/year), n = 569] [11]. We calculated the sample size by T-test on the assumption of a 15% improvement in the rate of renal deterioration, underα = 0.05, β = 0.2, intracluster GP number = 10. Assuming an intracluster correlation of 0.5 for safety, we obtained a study size of 2,264, estimating the proportion that would withdraw as 10%.

### Statistical analysis

Statistical analyses were performed using an intent-to-treat approach. For the proportion of absence from clinical visits of patients, and the co-treatment proportion between GPs and nephrologists, each proportion was analyzed by a generalized mixed-effect model of binary outcomes (binomial model) with intervention group as a fixed effect and clusters as a random effect. The deteriorating velocity of eGFR was analyzed by a linear mixed-effect model with intervention group as a fixed effect, and clusters and intercept of the line as a random effect.

For the analysis of clinical events, each comparison between group A and group B was conducted by a linear mixed-effect model with patient-nested cluster as the random effect. For comparison of the two groups, we adjusted for age, gender, presence of hypertension or diabetes, initial renal function, and geographical region. For comparison of eGFR between groups, to minimize the effect of missing values, we further performed comparisons with samples with more than 4 serum creatinine values. All of the statistical analyses were carried out using R version 3.0.1 software [12].

## Results

### Study clusters and patients

In the LMAs, 489 GPs recruited 2379 patients and obtained a complete baseline questionnaire and consent. The LMAs were randomly assigned to group A or group B (Fig 1). The characteristics of the patients in the two groups were similar at baseline, except for CKD stage and serum uric acid level. The proportion of cases of CKD stage 1+2 in group A was 49.4%, but it was 43.1% in group B, and the proportion of Stage 3 CKD in group A was 40.7%, but it was 47.4% in group B, while the average eGFR and average serum creatinine levels were identical in the two groups. Uric acid in group B was 6.25±1.67, while it was 6.08±1.48 in group A (Table 1).

**Table 1:** Characteristics of the patients at baseline.

	Group A	Group B	p value
number of clusters	23	26	
number of GPs	234	255	
number of GPs/cluster	10.17 ± 3.24	9.81 ± 3.16	
number of patients	1195	1184	
Male sex	71.1%	72.8%	0.39
Mean (SD) age (years)	63.17 ± 8.55	62.79 ± 8.25	0.26
CKD stages			0.01 *
1+2	49.43%	43.05%	
3	40.70%	47.44%	
4	8.35%	8.68%	
5	1.52%	0.82%	
TP (g/dl)	7.25 ± 0.52	7.23 ± 0.51	0.42
Alb (g/dl)	4.23 ± 0.39	4.24 ± 0.34	0.39
BUN (mg/dl)	19.31 ± 8.27	19.56 ± 7.60	0.48
s-cre (mg/dl)	1.08 ± 0.55	1.11 ± 0.52	0.30
eGFR (ml/min/1.73 m2)	59.72 ± 21.99	57.94 ± 22.05	0.06
proteinuria (g.cre)	0.63 ± 0.71	0.62 ± 0.68	0.74
BMI	25.85 ± 3.85	25.58 ± 3.95	0.10
Systolic BP	136.28 ± 13.44	136.71 ± 13.03	0.43
Diastolic BP	78.02 ± 9.33	78.40 ± 8.80	0.31
Presence of hypertension	90.29%	91.55%	0.32
anti-hypertensive medication	87.07%	88.29%	
HbA1c (%)	6.38 ± 1.21	6.31 ± 1.19	0.24
presence of DM	62.36%	60.29%	0.31
oral anti-diabetic medication	46.49%	45.74%	
uric acid (mg/dl)	6.08 ± 1.48	6.25 ± 1.67	0.02 *
presence of hyperuricemia	36.69%	41.11%	0.03 *
medication for hyperuricemia	21.19%	27.65%	
Hb (g/dl)	13.78 ± 1.78	13.74 ± 1.83	0.62
K (mEq/L)	4.40 ± 0.59	4.40 ± 0.57	0.99
HDL-C (mg/dl)	54.36 ± 16.58	54.01 ± 16.74	0.64
TC (mg/dl)	198.47 ± 36.71	195.96 ± 34.12	0.12
TG (mg/dl)	177.24 ± 141.14	176.94 ± 147.46	0.96
non-HDL-C (mg/dl)	144.23 ± 37.43	142.29 ± 34.46	0.26
Presence of hyperlipidemia	70.16%	67.18%	0.12
medication for dislipidemia (Statin)	39.73%	39.62%	

*;P<0.05.

### CKD treatment target Item changes

**Fig 2:**
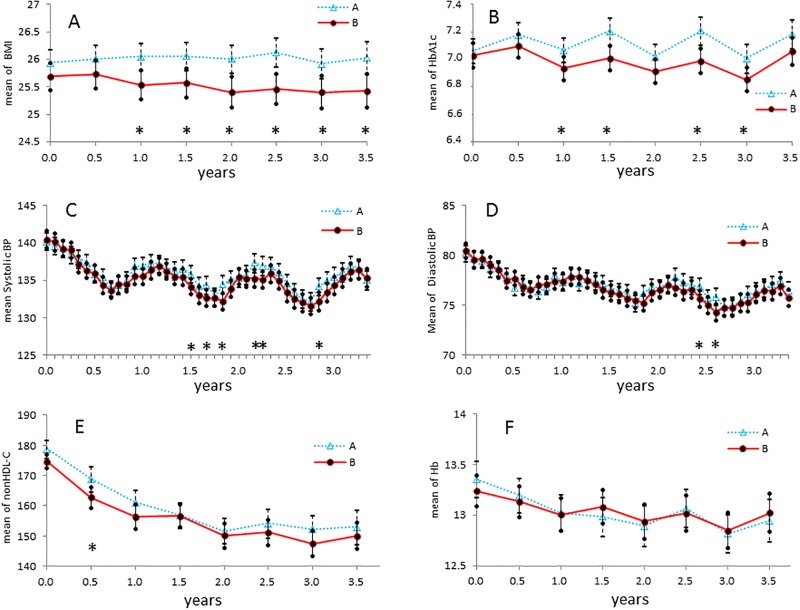
Effects of advanced CKD care system on CKD treatment targets. Items were BMI changes (A), HbA1c changes (B), systolic (C) and diastolic blood pressure changes (D), non-HDL cholesterol changes (E), and hemoglobin changes in subjects with Stage 3 CKD or later (F) during the study period. Patients in group B had greater improvements than those in group A in terms of the CKD practice guide targets, except for hemoglobin level. In particular, BMI, HbA1c, and blood pressure differences between group A and group B gradually increased over the time course of the interventions. Average BMI was significantly reduced in group B patients 2 years after starting the advanced intervention, and HbA1c was also significantly reduced in group B patients 2.5 years after starting the advanced intervention. * indicates p<0.05 between group A and group B by Student’s T test.

During the intervention period, 46% of patients in group B received 12 educational sessions, 73% of patients received more than 10 educational sessions, 11.2% of patients received fewer than 10 sessions, and 15.8% of patients stopped the educational intervention. Table 2 shows medication changes during the study period. In total, group B patients had greater improvements in CKD practice guide target items, except for hemoglobin level (Fig 2). With this intervention, there were gradually increasing differences in average values of each item between group A and group B. The average BMI difference between the 2 groups was significant after 2 years (Fig 2A). In addition, the average glycated hemoglobin difference was significant at 2.5 years (Fig 2B). While the percentage of medication of anti-diabetes drugs among group B decreased, that of group A increased. Although proportion of subject using insulin were increased in both groups, obvious increment was observed in group A (Table 2 and S3 Table). Blood pressure of the two groups was identical at randomization, and remained unchanged during the study (Fig 2C and 2D). The percentages of medication of anti-hypertensives and any class of anti-hypertensive drugs were identical between the groups (Table 2 and S4 Table).

**Table 2:** Percnetage of Medication of various drugs and their changes during the study.

Study period	anti-hypertensives(%)		oral anti-DM (%)		anti-hyperuricemia (%)		Statin (%)		ESA (%)	
years	Group A	Group B	Group A	Group B	Group A	Group B	Group A	Group B	Group A	Group B
0.0	87.07	88.29	46.49	45.74	21.19	27.65	39.73	39.62	1.20	0.60
0.5	89.62	90.59	46.35	45.79	22.96	29.46	41.51	40.99	1.14	0.98
1.0	91.22	90.73	46.16	45.69	24.86	30.62	44.88	42.60	1.46	1.03
1.5	91.81	91.54	46.14	45.82	25.80	32.30	46.52	43.39	1.69	0.97
2.0	90.50	91.05	46.72	45.73	27.13	34.19	46.23	43.14	2.06	1.29
2.5	88.13	88.31	47.19	46.05	27.43	35.18	46.78	43.38	2.15	1.33
3.0	87.96	87.43	47.08	45.12	27.25	35.72	46.72	42.77	1.95	1.41

DM; diabetes mellitus.

### Primary outcomes

#### 1) Rate of absence of patients from clinical visits

There were 193 patients in group A and 136 patients in group B who were absent from regular clinical visits for more than 6 months. Table 3 shows the rates of absence from clinical visits among the subjects. A total of 16.2% of group A subjects and 11.5% of group B subjects ceased regular visits for more than 6 months. These figures were significantly different (P = 0.01) after adjustment for age, gender, presence of hypertension or diabetes, initial renal function, and geographical region.

**Table 3:** Primary outcomes.

endpoint	Group A	Group B	p value
primary assesement factors			
The rate of discontinuous clinic visits			
No. of events	193	136	
crude rate	16.2%	11.6%	0.01#1*
Co-treatment between GPs and nephrologists			
subjects reaching the criteria for referral	976	904	
referred	165	289	
rate	16.9%	32.0%	<0.01#1**
reintroduced to GP	89	195	
rate	9.1%	21.6%	<0.01#1**
eGFR deterioration speed differences (ml/min/1.73 m^2^)			
CKD stages	average trend of yearly eGFR changes SD	average trend of yearly eGFR changes SD	p value
total subjects #2			
All stages	-2.60 ± 5.8	-2.41 ± 5.08	0.07
CKD Stage 1+2	-2.84 ± 5.98	-2.87 ± 5.78	0.78
CKD Stage 3	-2.42 ± 5.93	-1.93 ± 4.41	0.03
CKD Stage 4	-2.72 ± 3.76	-3.08 ± 3.48	0.26
CKD Stage 5	-2.00 ± 1.43	-3.79 ± 3.27	0.44
subjects with more than 4 creatinine records #2			
All stages	-2.36 ± 3.88	-2.17 ± 3.52	0.07
CKD Stage 1+2	-2.63 ± 4.29	-2.53 ± 4.16	0.66
CKD Stage 3	-2.22 ± 3.76	-1.83 ± 3.04	0.04
CKD Stage 4	-2.42 ± 2.52	-2.46 ± 2.19	0.29
CKD Stage 5	-2.02 ± 1.48	-2.16 ± 1.47	0.56

^#1^ For the primary outcome analysis, we adjusted with age, gender, presence of hypertension or diabetes, initial renal function, and geographical region.

^#2^ Each CKD stage analysis was separately done.

*;P<0.05

**;P<0.01.

During the study period, an absence from consultation for 2 months or more in group B subjects was observed 1255 times (see S2 Table). A significantly higher cessation rate of regular visits was observed in male subjects in group A (group A: male 18.5%, female 10.5%; group B: male 11.7%, female 11.2%). The main reason for the difference between the groups was the significant improvement of visits by males in group B via support from the coordinating center.

#### 2) Co-treatment rate between GPs and nephrologists

During the entire period, 976patients in group A and 904 patients in group B reached the criteria for requiring nephrology care, and 165 patients (16.9%) in group A and 289 patients (32.0%) in group B were introduced to nephrologists. A significantly higher referral rate was observed in group B after adjustment for age, gender, presence of hypertension or diabetes, initial renal function, and geographical region (P<0.01). Among 976 patients who reached the criteria for requiring nephrology in Group A, 240 were due to a ratio of urinary protein/urinary creatinine ≥ 0.5 or proteinuria ≥ 2+, 244 patients were due to both proteinuria and hematuria being positive, 130 patients showed estimated GFR (eGFR) < 50 mL/min/1.73 m^2^, and 171 patients had both urinalysis abnormalities and eGFR exacerbation, Among 904 patients who reached the criteria for requiring nephrology in Group B, 238 patients were due to a ratio of urinary protein/urinary creatinine ≥ 0.5 or proteinuria ≥ 2+, 135 patients were due to both proteinuria and hematuria are positive (≥ 1+), 179 patients showed eGFR < 50 mL/min/1.73 m^2^, and 138 patients had both urinalysis abnormalities and eGFR exacerbation.

In Group A subjects who reached the criteria for requiring nephrology care, 8.5% of subjects with urinary abnormalities, 13.9% of subjects with eGFR exacerbation and 29.3% having both criteria were introduced to nephrologists. In Group B subjects who reached the criteria for requiring nephrology care, 21.4% of subjects with urinary abnormalities, 42,5% of subjects with eGFR exacerbation and 37.8% with both criteria were introduced to nephrologists. Among the subjects who reached the criteria for requiring nephrology care, there was a significant initial eGFR difference between referred subjects and non-referred ones (group A: referred 44.6±23.5 ml/min/1.73 m^2^, non-referred 59.5±20.4 ml/min/1.73 m^2^; group B: 50.4±21.0 ml/min/1.73 m^2^, 56.6±20.9 ml/min/1.73 m^2^).

Among the referred subjects, 89 patients in group A and 195 patients in group B were reintroduced to GPs, and a significantly higher co-treatment rate was observed in group B (P<0.01). Comparing the reintroduced subjects with the non-reintroduced ones, initial eGFR was significantly higher in the former group in group A, while this difference was not observed in group B (group A: reintroduced 49.3±25.4 ml/min/1.73 m^2^, not reintroduced 38.3±18.9 ml/min/1.73 m^2^; group B: 49.9±20.2 ml/min/1.73 m^2^, 51.1±22.2 ml/min/1.73 m^2^).

#### 3) progression of CKD stages

There was no significant difference between groups in the rate of eGFR deterioration. (group A: 2.6±5.8 ml/min/1.73 m^2^, group B: 2.4±5.1 ml/min/1.73 m^2^, p = 0.07). There was a significant difference in the eGFR deterioration rate in subjects with Stage 3 CKD (group A: 2.4±5.9 ml/min/1.73 m^2^, group B: 1.9±4.4 ml/min/1.73 m^2^, p = 0.03), while the eGFR deterioration rates were identical for the other CKD stages. Each CKD stage analysis was conducted separately.

We compared eGFR deterioration rates using linear regression, and to minimize sampling bias we further analyzed subjects who had more than 4 records of serum creatinine. As shown in Table 3, the differences in eGFR deterioration rate were the same as in the analysis of total subjects.

### Secondary outcomes

**Fig 3:**
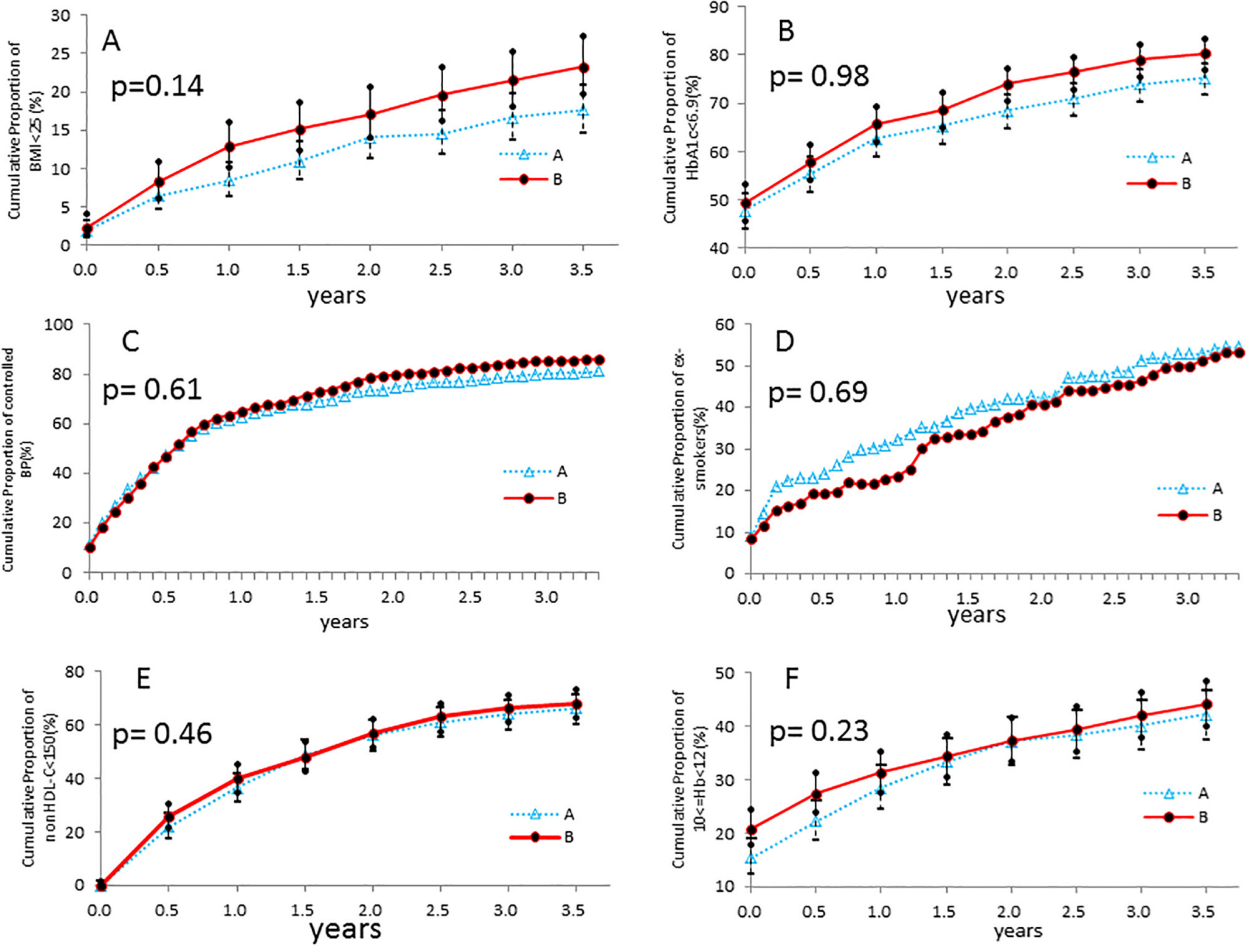
CKD treatment control achievements during the study period. CKD treatment cumulative achievements for BMI <25 (A), HbA1c <6.9% (B), controlled blood pressure (C), smoking cessation (D), non-HDL-C <150 mg/dl (E), and proportion of hemoglobin concentration controlled to 10–12 g/dl among CKD stages 3, 4, and 5 (F). There was no significant difference between group A and group B in CKD treatment control achievements by generalized linear mixed model. Patients in group B had greater cumulative incidences of achieving the targets of BMI <25, glycated hemoglobin <6.9%, and other measured risk factors, except for non-HDL cholesterol level and smoking cessation rate. In particular, cumulative differences in the rates of achieving BMI, HbA1c, and blood pressure targets between group A and group B gradually widened over the course of the interventions.

**Fig 4:**
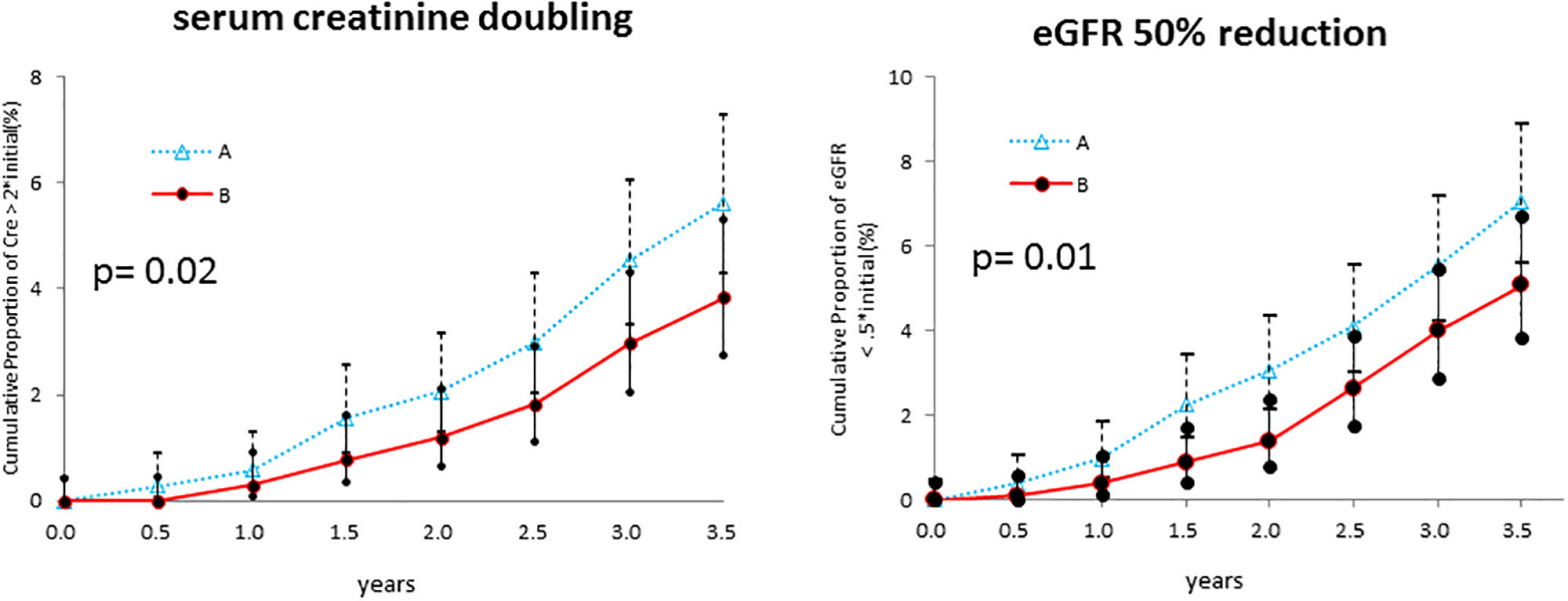
Renal function outcomes. Both the number of subjects with a doubling of serum creatinine and the number with a 50% reduction in eGFR significantly decreased in group B. The cumulative incidence of a doubling of serum creatinine or a 50% reduction in eGFR also exhibited a gradually increasing difference between groups A and B over the course of the study. A significant difference was shown between Group A and Group B by generalized linear mixed model.

There was no significant difference between group A and group B in terms of the cumulative incidence of achieving the CKD treatment guide targets at the end of study (Table 4). However, during the study period, more patients in group B had reached the targets of BMI <25, glycated hemoglobin <6.9%, and other measured risk factors, except for non-HDL cholesterol level and smoking cessation rate, and the differences between group A and B became wider in BMI, HbA1c, and blood pressure with time after the start of the study (Fig 3). The numbers of subjects with a 50% reduction in urinary protein were identical; however, the number of subjects with a doubling of serum creatinine or 50% reduction in eGFR was significantly reduced in group B. The cumulative incidence of a doubling of serum creatinine or 50% reduction in eGFR also showed a gradually increasing difference between groups A and B over the course of the study. Finally, 6.7% of group A subjects and 4.4% of group B subjects showed doubling of serum creatinine (P = 0.02) and 8.1% of group A subjects and 5.8% of group B subjects showed 50% reduction of eGFR (P = 0.01) at 3.5 years, which were significantly different between the groups by the generalized linear mixed model (Table 4 and Fig 4). However, the cumulative incidences of patients starting renal replacement therapy and the incidences of cardiovascular events were identical in the two groups (Table 4).

**Table 4:** Proportion of adherence to the complete CKD treatment guide.

		Group A		Group B		p value
CKD practice guide item	target	n	%	n	%	
number of subjects		1195		1184		
Smoking	number at risk, %	196	16.4%	189	16.0%	
	cessation of smoking, N, %	106	54.1%	96	50.8%	0.69
BMI>25	number at risk, %	641	53.6%	567	47.9%	NS
	reduced to less than 25,n, %	115	17.9%	125	22.0%	0.14
Systolic BP>130 and/or diastolic BP>80	number at risk, %	869	72.7%	874	73.8%	
	controlled to target level	716	82.4%	739	84.6%	0.56
HbA1c>6.9%	number at risk, %	732	61.3%	700	59.1%	
	controlled to less than 6.9%, N, %	550	75.1%	543	77.6%	0.98
Non-HDL cholesterol >150 mg/dl	number at risk, %	356	29.8%	370	31.3%	
	controlled to less than 150 mg/dl	228	64.0%	246	66.5%	0.46
CKD stage 3,4,5	number at risk, %	520	43.5%	618	52.2%	
	controlled hemoglobin concentration to 10–12 g/dl	222	42.7%	277	44.8%	0.23
Blood pressure measurement> 14 days/month	number at risk, %	1017	85.1%	975	82.3%	
Blood pressure measurement> 14 days/month		972	95.6%	933	95.7%	0.35
Proteinuria	number at risk, %	358	30.0%	481	40.6%	
subjects with 50% reduction in urinary protein		149	41.6%	233	48.4%	0.13
serum creatinine doubling		71	6.7%	48	4.4%	0.02*
eGFR 50% reduction		85	8.1%	61	5.8%	0.01*
incidence of cardiovascular events		79	7.5%	67	6.4%	0.35
incidence of starting RRT		23	2.2%	24	2.3%	

For calculation of p value, we applied generalized linear mixed model.

*;P<0.05.

## Discussion

In general, poor adherence to medication is associated with the development of complications, disease progression, avoidable hospitalizations, higher medical costs, premature disability, and death[13],[14]. Discontinuous clinical visit rates are relatively high across disease states, treatment regimens, and age groups in males, with the first several months of therapy characterized by the highest rate of discontinuation [5]. Most CKD patients are asymptomatic, and discontinuous clinical visit is one of the main problems of such patients. In this study, our intervention methods successfully achieved behavior modification of CKD patients by producing significantly more regular clinical visits in Group B. Not only encouragement to make regular clinical visits, but also regular educational sessions by dieticians and entering clinical studies with consent may have had some effect in this study. Meanwhile, the discontinuous clinical visit rates in female subjects were identical between the two groups. For the avoidance of discontinuous clinical visits, this type of intervention was thus not effective in female subjects. There are several explanations for this gender differences. One is that males often received regular educational sessions from dieticians with their family, especially with their wife, while females often received the sessions alone. Males had more chances to hear advice from their families than females. Furthermore, support provided by females was positively related to patients' intention to adhere, whereas support provided by males was slightly negatively related to the intention of their female spouses to adhere [15].

Appropriate timing of referral to nephrologists has been shown to delay the progression of CKD,[16]. In this study, we successfully observed behavior modification of GPs by our group B interventions. On the basis of the Japanese Clinical Practice Guidebook for Diagnosis and Treatment of Chronic Kidney Disease [17], we encouraged referral to a nephrologist at eGFR <50 ml/min/1.73 m^2^ or upon overt proteinuria. However, most of our subjects were not referred due to overt proteinuria. Proteinuria was shown to be the most important risk factor for the progression of ESRD [6], but there was no evidence of clinical improvement by such early referral to a nephrologist. We will continue to follow-up our subjects and will show the effect of appropriate referral to a nephrologist on the prognosis of early-stage CKD patients in the near future.

Several previous studies showed that concentrated educational interventions in the early period of the trial had a good effect on the rate of renal function deterioration and patient outcome [18] [19]. In this study, the educational interventions in group B were 30-minute sessions every 3 months during the entire intervention period, the effects of which would have appeared gradually. As shown in Figs 2 and 3, except for non-HDL cholesterol concentration and hemoglobin changes, subjects in group B showed gradual improvements of glucose control, and body weight control compared with group A patients. Those changes might be due to the multifactorial intervention in Group B. As a result, significant differences in creatinine doubling and eGFR 50% reduction were observed, and the differences between groups A and B widened over time (Fig 4). In addition, the average rate of eGFR deterioration tended to be lower in group B, while the medication rates of ARB and ACEI were identical between the groups.

A favorable effect in this study was obvious in subjects with Stage 3 CKD. For evaluation of renal function, we used a serum creatinine-based GFR estimation formula for the Japanese. This formula is suitable for predicting renal function of less than GFR <60 ml/min/1.73 m^2^ [20]. Consequently, estimation of GFR with this eGFR formula had a wide range of potential over- and underestimation in CKD stages 1 and 2. Meanwhile, in stage 3 subjects, a significant improvement of eGFR deterioration rate was observed. Stage 3 CKD is the most prevalent in the CKD population [21],[22], and most such patients were cared for by GPs. Meanwhile, in CKD stage 4 and 5 patients, no favorable effect was observed. Recent trials for advanced CKD patients did not show favorable effects on renal outcome at CKD stages 4 and 5 [23–25]. Several reports suggested that predialysis nephrologist care might have favorable effects on patient outcomes in those stages of CKD upon receiving multidisciplinary treatment [19,26]. We have to determine the ideal treatment methods for CKD stage 4 or 5, in order to effectively delay the progression of renal function deterioration.

Finally, a limitation of this study was that we could not observe any differences in the cumulative incidence of patients starting renal replacement therapy or suffering cardiovascular events between the two groups, but we think this type of intervention would have an effect on the incidences of RRT and CVD events after long-term observation. Several previous studies showed that such educational intervention had a long-term legacy effect [27,28]. We have to obtain longer follow-up results after intervention in the near future. Furthermore, several follow-up studies of CKD patients at annual screening in the general population [29] or in nephrology clinics [30] [31] [32] have been performed, but there was only one report about long-term follow-up studies of CKD patients cared for by GPs [33]. There are few interventional clinical studies of CKD patients cared for by GPs, and there is also little information about the detailed clinical outcome and treatment pattern changes in recent years at GPs. A long-term follow-up of this subject would be also valuable.

In conclusion, an advanced CKD care system could achieve behavior modification of CKD patients, namely, significantly lower discontinuous clinical visit rates, and behavior modification of both GPs and nephrologists, namely, significantly higher referral and co-treatment rates, resulting in the retardation of CKD progression, especially in patients with proteinuric Stage 3 CKD. For the purpose of lifestyle modification for preventing the progression of kidney dysfunction and complications, this type of multifactorial intervention might be one solution.

## Supporting Information

S1 Fig Distribution of clinical sites in Japan. (DOCX)

S2 Fig Comprehensive lifestyle and nutrition education score sheet. (TIFF)

S1 Protocol (DOC)

S1 Table CKD treatment goals in this study. (DOCX)

S2 Table Number and detailed contact methods for group B patients who did not receive regular consultation at GPs for 2 months and over. (DOCX)

S3 Table Detail of anti-diabetes drug changes during the study period. (DOCX)

S4 Table Detail of anti-hypertensive drug changes during the study period. (DOCX)
