# Supplementary material for: Effect of Behavior Modification on Outcome in Early- to Moderate-Stage Chronic Kidney Disease: A Cluster-Randomized Trial
Source: PLoS One. 2016 Mar 21;11(3):e0151422. doi: 10.1371/journal.pone.0151422 (PMC4801411; doi:10.1371/journal.pone.0151422)
Supplement: S1 Fig — (DOCX) [file pone.0151422.s001.docx]

**S1 Fig. Distribution of clinical sites.**

We recruited 49 local medical associations (clusters) in 15 different prefectures, which were classified into our regions (strata) based on the level of increase in the rate of dialysis patients.

**
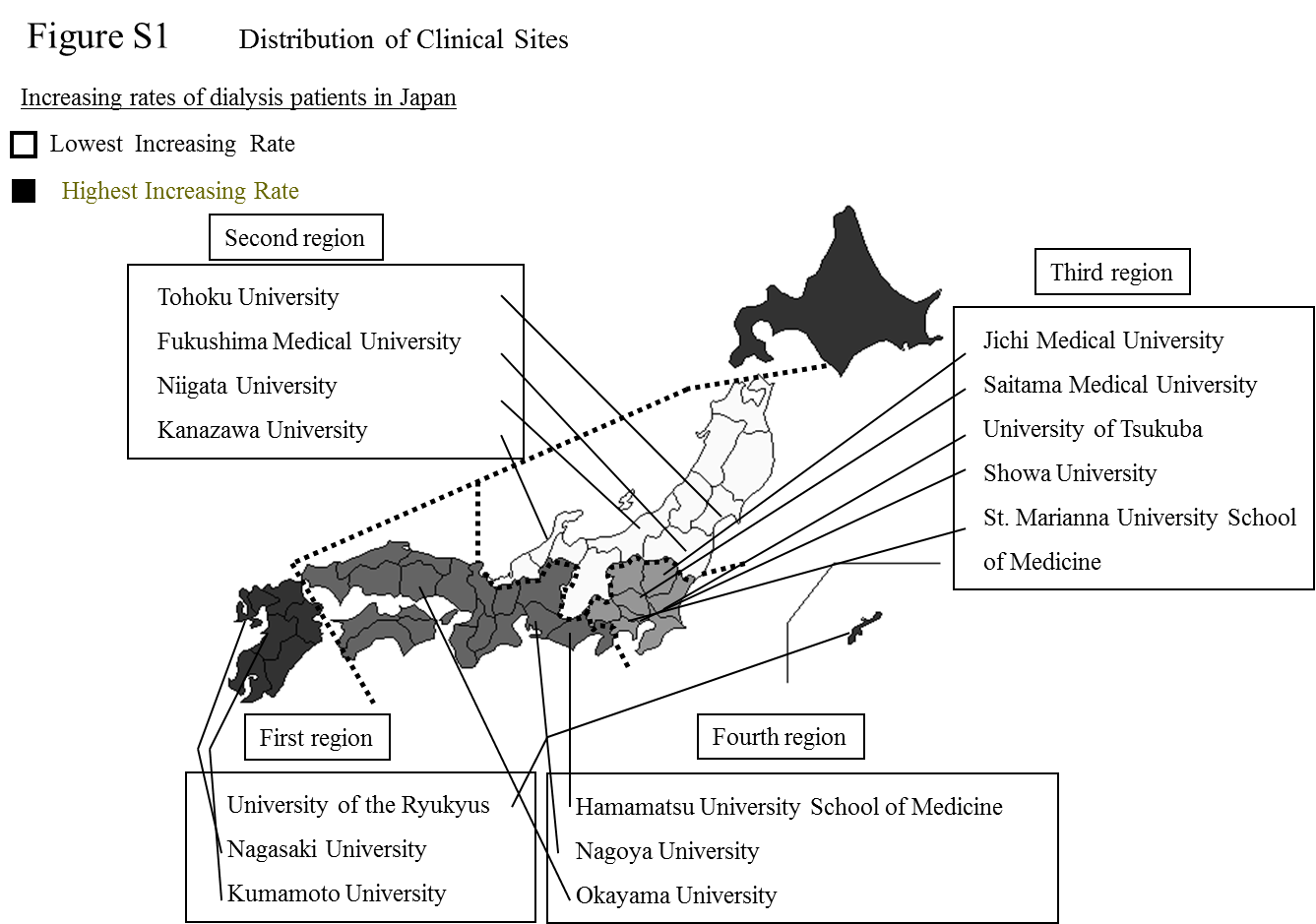
**
