# Supplementary figures and images for: Effect of Behavior Modification on Outcome in Early- to Moderate-Stage Chronic Kidney Disease: A Cluster-Randomized Trial
Source: PLoS One. 2016 Mar 21;11(3):e0151422. doi: 10.1371/journal.pone.0151422 (PMC4801411; doi:10.1371/journal.pone.0151422)

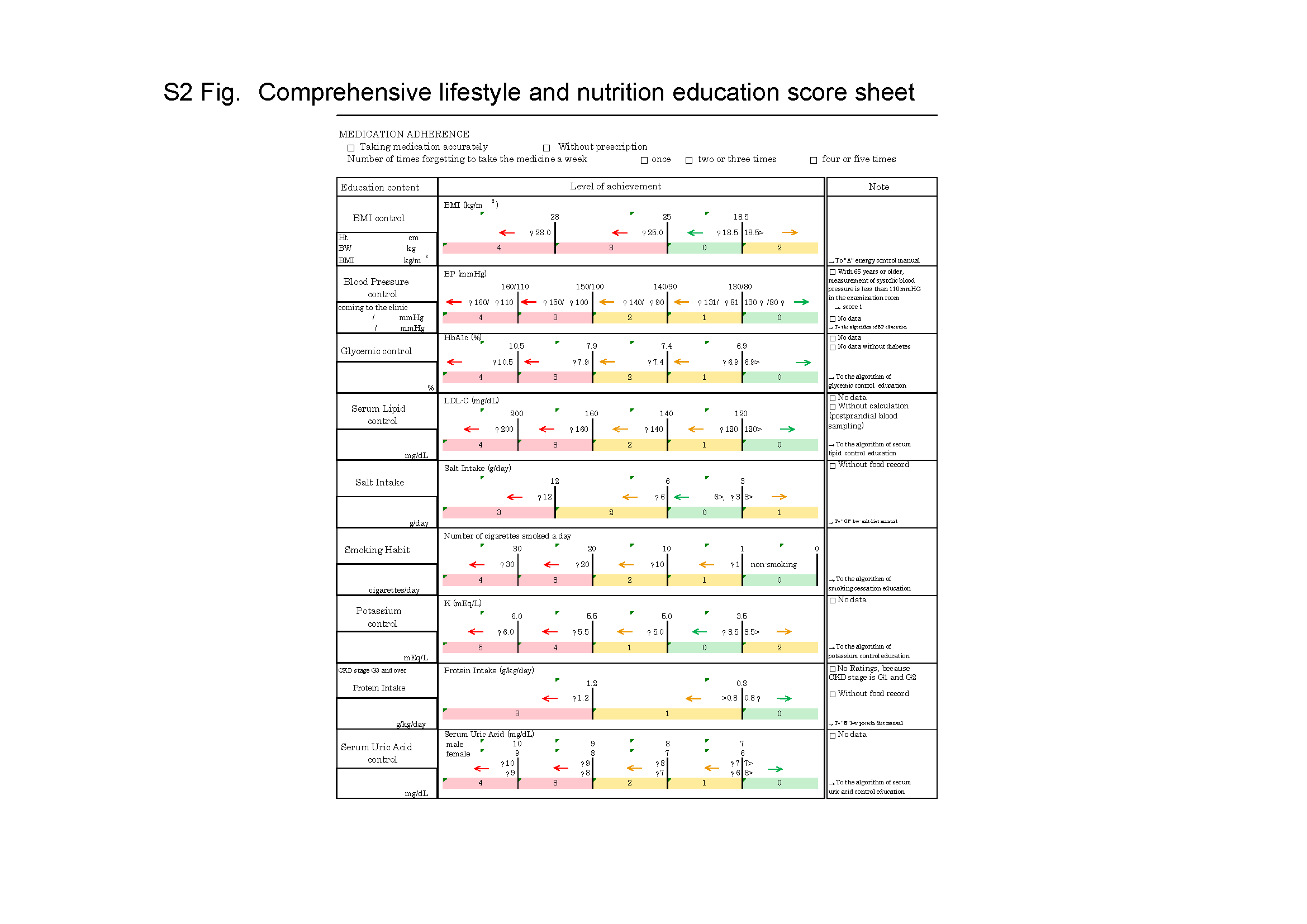

Supplement: S2 Fig — (TIFF) [file pone.0151422.s002.tiff]
