# Supplementary material for: Effect of Behavior Modification on Outcome in Early- to Moderate-Stage Chronic Kidney Disease: A Cluster-Randomized Trial
Source: PLoS One. 2016 Mar 21;11(3):e0151422. doi: 10.1371/journal.pone.0151422 (PMC4801411; doi:10.1371/journal.pone.0151422)
Supplement: S1 Protocol — (DOC) [file pone.0151422.s003.doc]

A study to examine the utility of

the medical examination and treatment system

by the cooperation of the general physician and nephrologist for the prevention of the aggravation chronic kidney disease

Study protocol

The Kidney Foundation, Japan

The first edition: Feb 6, 2008

# Summary

# 1. Hypothesis of the study

1. Clinical practice in accordance with the Japanese CKD Clinical Practice Guide will improve the prognosis of CKD patients and reduce the speed of renal function deterioration.

2. Education-based interventions for CKD patients by registered dietitians and other co-medicals will help achieve strict CKD treatment goals in accordance with the Japanese CKD Clinical Practice Guide.

3. Collaboration concerning clinical practices among general physicians, nephrologists, and co-medicals will reduce the gap between clinical practice and evidence-based care measures, and improve the rate of continued consultation and prognosis in CKD patients.

4. These active interventions to improve CKD treatment will achieve the desired effects in terms of medicoeconomics

## 2. Study design

The study was a stratified open cluster-randomized study with two intervention groups.

Since the increase in the rate of dialysis patients varies from region to region in Japan [Usami T, et al: JAMA. 2000; 284: 2622–4.], we divided the country into four regions (Fig. 1) as strata, so that they would include at least one managing facility and two or more clusters.


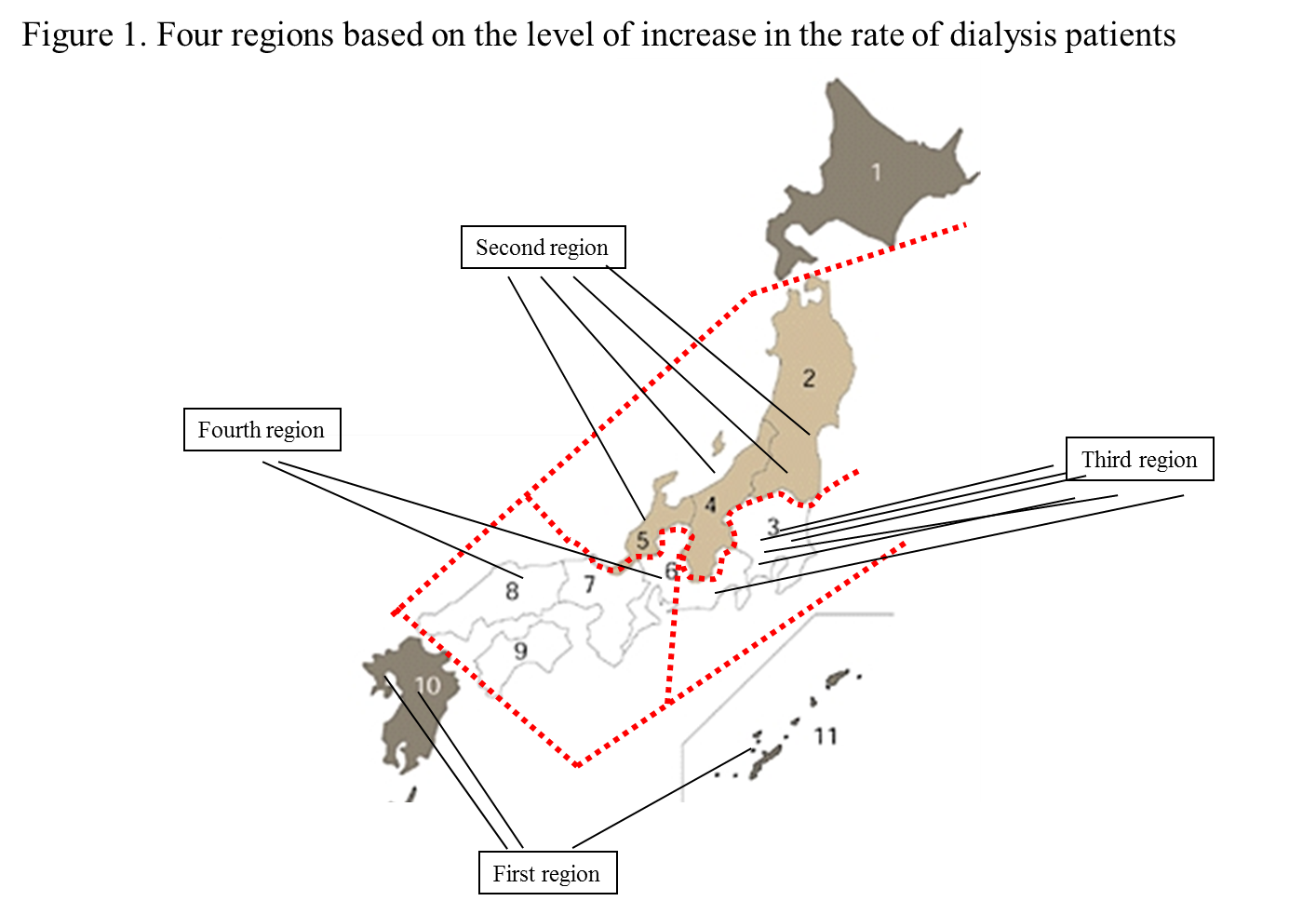


(1) Recruitment of managing institution

In this study, we recruit public participation for managing institutions more than 8 in the whole country. The managing institution cooperate local medical association and nephrologist. We recruit the managing institutions of minimum one institution from all district regions (Fig. 1).

(2) Selection of local medical association

Managing institution selects local medical associations eligible for all the following conditions.

- The cooperation with the nephrologist is provided.
- Around 10 general physicians can anticipate registration.
- The cooperation system between general physicians and nephrologists is established.
- There is little interchange between local medical associations

(3) Selection of nephrologist

The managing institution selects nephrologist who can participates this study. The list of nephrologists is showed to the local medical associations. The nephrologists must be attends the lecture about this study.

(4) Recruitment of general physician

The local medical association recruits around 10 general physicians eligible for all the following conditions from April 1, 2008 to June 30, 2008.

- Who belongs in each local medical association
- Who is not nephrologist
- Around 5 CKD patients can register.

(5) Recruitment of participant

General physician recruits participants who are eligible this study from April 1, 2008 to September 30, 2008. After informed consent is obtained from participant, general physician registers participant to data center. The aim of the number of the participants is each 1,250 in groups, 2,500 in total. When the number of the participants does not reach the aim by September, 2008, the registration of the participant continues until October, 2008.

(6) Assignment and randomization

This study is a stratified open cluster-randomized study with two intervention groups: group A (standard intervention) and group B (advanced intervention). Each local medical association was regarded as a cluster. For randomization, we divided the country into four regions as strata because the rate of increase of dialysis patients varies from region to region in Japan (Fig 1). All general physicians in each local medical association are assigned to the same intervention group, and do not change the intervention method during this study.

(7) Period of treatment

The period of treatment is from October, 2008 to March, 2012.

(8) Intervention methods

Participants in group A clusters are instructed to undergo treatment in accordance with the current CKD treatment guide only.

Whereas participants in group B clusters are not only instructed in the same method but also receive service by the coordinating center and educational sessions from dieticians upon visiting their local general physician’s offices every three months. The centers contacted patients by letter, telephone, or email a week before the consultation day, and encouraged those who had not consulted a physician for over two months to receive care, trying to prevent their withdrawal from treatment. To facilitate referrals to nephrologists, the center sent a list of patients who met the criteria for referral to the physicians and clinical research coordinators. In addition, the coordinating center closely monitors the treatment status and provides the group B general practice office with comments on the data.

(9) Subcohort Analysis

Subcohort analysis is carried at a part of participants.

## 3. Inclusion criteria for participant

Who attends to general physician

Age between 40 and 74 years

Chronic kidney disease（CKD）stage 1，2，4，or 5, or CKD stage 3 with proteinuria and diabetes or hypertension.

## 4. Exclusion criteria for participant

Dialysis patients

Patients who undergoes treatment of CKD only at nephrologists, not at general practical physicians

Patients who undergoes treatment of CKD at physicians who do not register this study

## 5. Method of Treatment

### 5.1 Goals for the treatment of CKD

Participants in the study, or patients, will receive treatment according to the CKD Clinical Practice Guide (Table 1, CKD Clinical Practice Guidelines: Japanese Society of Nephrology (written and edited), First edition, Tokyo Igakusha, 2007).

・lifestyle modifications to avoid obesity and stop smoking are necessary.

・Sodium restriction is less than 6 g/day.

・Protein restriction is 0.6～0.8 g/kg/day in CKD stage 3,4 and 5.

・Strict blood pressure control (less than 130/80 mmHg), if proteinuria is over 1g/gCr, more strict blood pressure control (less than 125/75 mmHg) is needed. To control blood pressure, use of renin-angiotensin system inhibitors is favorable, and, if necessary, addition of the other antihypertensive drug is recommended.

・ Strict blood sugar control (HbA1c <6.5%)

・Low-density lipoprotein (LDL)-cholesterol control (LDL-C<120 mg/dl)

・ Anemia control (Hb: not less than 10g/dL and less than 12g/dL)


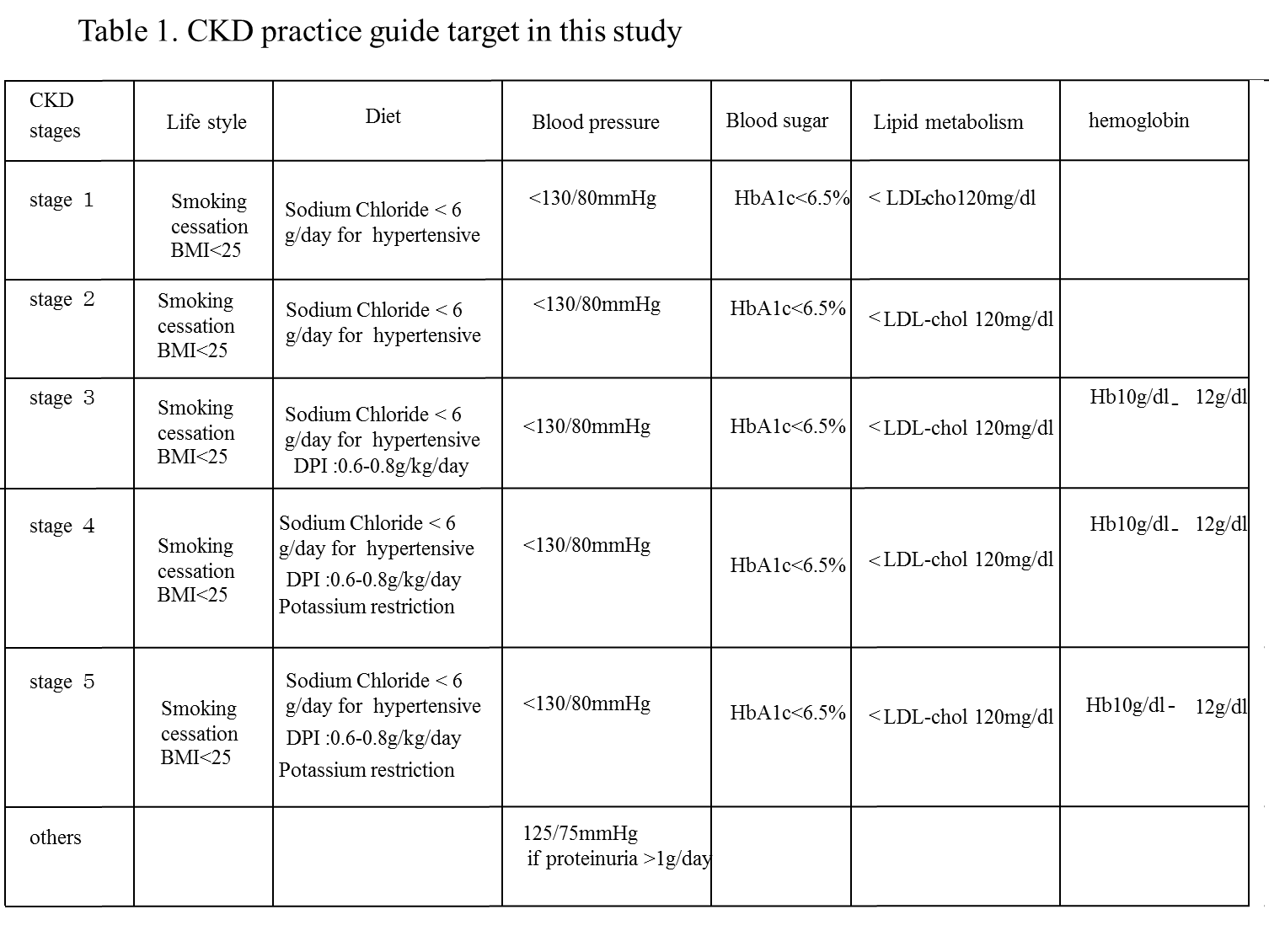


### 5.2 Criteria for referral from general physician to nephrologist

If participant is eligible for following conditions, general physician refer the participant to nephrologist.

・Ratio of urinary protein/urinary creatinine ≥ 0.5 or proteinuria ≥ 2+;

・Estimated GFR (eGFR) < 50 mL/min/1.73 m^2^

・Both proteinuria and hematuria are positive (≥ 1+)

・When general physicians judge that patients should consult a nephrologist

eGFRs in this study were calculated using the following formula:

eGFR (mL/min/1.73 m^2^)=194 x Age^-0.287^ x Cre^-1.094^ (x 0.739 in the case of women)

## 6. Data collection

At consultation to general physician, data are collected as follows (Table 2)

Table 2. Schedule of data collection


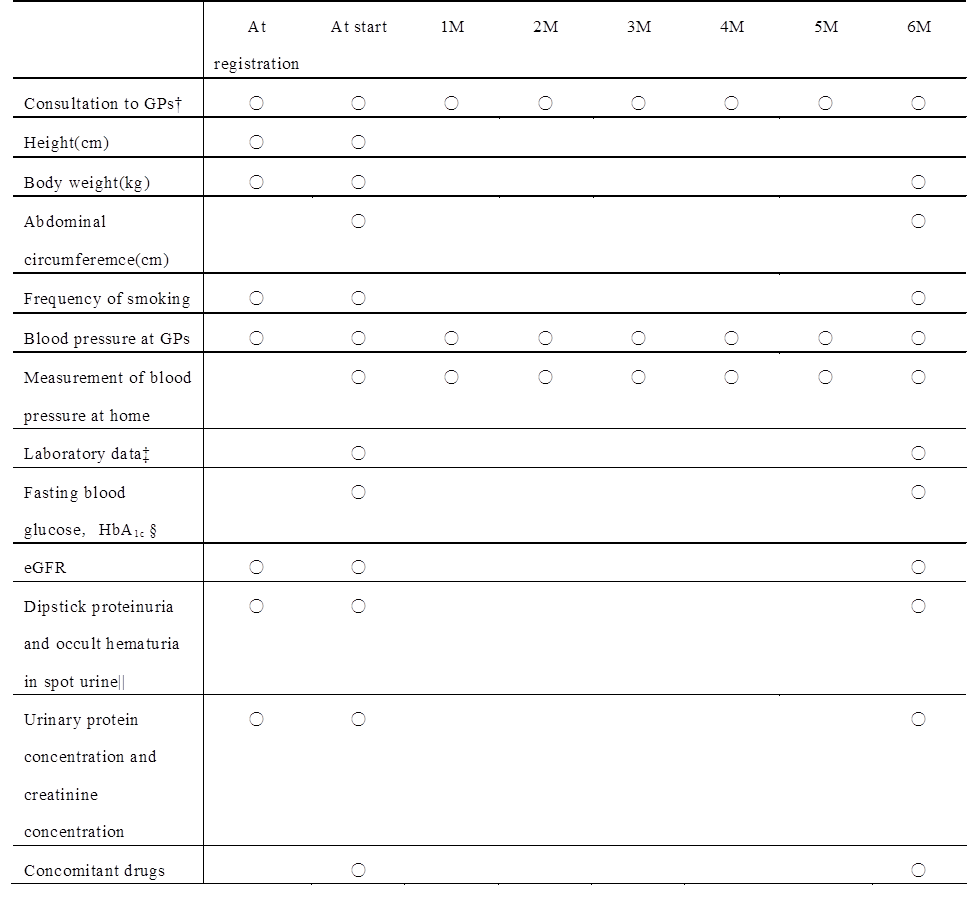


* Schedule repeats after 7 months onward until the end of the study

†In principal, participants consult to GPs once a month.

‡ Serum creatinine，BUN，Potassium，Hb，HDL-C，TC，TG，Urine Acid，TP，Alb（at fast, in principal）

§ Only in diabetic participants

|| At registration, only dipstick proteinuria is needed.

## 7. Sample size

Managing institution: more than 8 in whole country

Local medical association: more than 49 in whole country

General physician: around 10 in each local association

Participant: around 5 in each general physician

Target number of participants is 2,500 (1,250 for each group).

## 8. Assesment

## 8.1 Primary endpoint

1. The rate of continuous clinic visits of participants

2. The proportion of patients under cotreatment between general physicians and nephrologists

1) The rate of reference from general physician to nephrologist

2) The rate of reference from nephrologist to general physician

3. Annual changes in CKD stage

To design the sample size of participant in this study, annual change of GFR is used instead of annual change in CKD stage.

## 8.2 Secondary endpoint

1. The proportion of adherence to the complete CKD treatment guide

2. The rate of achievement of blood pressure goals

3. The number of participants with 59% reduction in urine protein

4. The number of participants with a doubling of serum creatinine or 50% reduction in eGFR

5. Yearly changes in the number of patients starting renal replacement therapy

6. The incidence of cardiovascular events

# 9. System of study

# 9.1 Principal Investigator

Osamu Sakai

Chairman of the Board of Directors

The Kidney Foundation, Japan

# 9.2 Leader of the study

Kunihiro Yamagata

Professor

Pathophysiology of Renal Diseases, Graduate School of Comprehensive Human Sciences, University of Tsukuba

# 
