# Supplementary material for: Effect of Behavior Modification on Outcome in Early- to Moderate-Stage Chronic Kidney Disease: A Cluster-Randomized Trial
Source: PLoS One. 2016 Mar 21;11(3):e0151422. doi: 10.1371/journal.pone.0151422 (PMC4801411; doi:10.1371/journal.pone.0151422)
Supplement: S1 Table — (DOCX) [file pone.0151422.s004.docx]

| S1 Table. CKD practice guide target in this study | | |  |  |  |  |
| --- | --- | --- | --- | --- | --- | --- |
|  |  |  |  |  |  |  |
| CKD stages | Lifestyle | Diet | Blood pressure | Blood sugar | Lipid metabolism | hemoglobin |
| Stage 1 | Smoking cessation BMI<25 | Sodium Chloride < 6 g/day for hypertensive | <130/80mmHg | HbA1c<6.9% | Non-HDLcho<150mg/dl |  |
| stage 2 | Smoking cessation BMI<25 | Sodium Chloride < 6 g/day for hypertensive | <130/80mmHg | HbA1c<6.9% | Non-HDLcho<150mg/dl |  |
| stage 3 | Smoking cessation BMI<25 | Sodium Chloride < 6 g/day for hypertensive DPI :0.6-0.8g/kg/day | <130/80mmHg | HbA1c<6.9% | Non-HDLcho<150mg/dl | Hb10g/dl-12g/dl |
| stage 4 | Smoking cessation BMI<25 | Sodium Chloride < 6 g/day for hypertensive DPI :0.6-0.8g/kg/day Potassium restriction | <130/80mmHg | HbA1c<6.9% | Non-HDLcho<150mg/dl | Hb10g/dl-12g/dl |
| stage 5 | Smoking cessation BMI<25 | Sodium Chloride < 6 g/day for hypertensive DPI :0.6-0.8g/kg/day Potassium restriction | <130/80mmHg | HbA1c<6.9% | Non-HDLcho<150mg/dl | Hb10g/dl-12g/dl |
| others |  |  | <125/75mmHg  if proteinuria >1g/day |  |  |  |
|  |  |  |  |  |  |  |
