# Supplementary material for: Effect of Behavior Modification on Outcome in Early- to Moderate-Stage Chronic Kidney Disease: A Cluster-Randomized Trial
Source: PLoS One. 2016 Mar 21;11(3):e0151422. doi: 10.1371/journal.pone.0151422 (PMC4801411; doi:10.1371/journal.pone.0151422)
Supplement: S2 Table — (DOCX) [file pone.0151422.s005.docx]

S2 Table. Number and detailed contact methods for group B patients who did not receive regular consultation at GPs for 2 months and over

| Method | letter | phone call | e-mail | Total |
| --- | --- | --- | --- | --- |
| number | 982 | 232 | 41 | 1255 |
