# Supplementary material for: Effect of Behavior Modification on Outcome in Early- to Moderate-Stage Chronic Kidney Disease: A Cluster-Randomized Trial
Source: PLoS One. 2016 Mar 21;11(3):e0151422. doi: 10.1371/journal.pone.0151422 (PMC4801411; doi:10.1371/journal.pone.0151422)
Supplement: S3 Table — (DOCX) [file pone.0151422.s006.docx]

| S3 Table | Detail of anti-diabetes drugs changes during study period | | | | | | | |  |  |  |  |  |  |  |  |  |  |  |  |  |
| --- | --- | --- | --- | --- | --- | --- | --- | --- | --- | --- | --- | --- | --- | --- | --- | --- | --- | --- | --- | --- | --- |
|  |  |  |  |  |  |  |  |  |  |  |  |  |  |  |  |  |  |  |  |  |  |
| Study period | | insulin | |  | DPP-4 Inhibitors | |  | Sulfonylureas | |  | Alpha-glucosidase　Inhibitors | |  | Meglitinides | |  | Thiazolidinediones | |  | Biguanides | |
| years |  | Group A | Group B |  | Group A | Group B |  | Group A | Group B |  | Group A | Group B |  | Group A | Group B |  | Group A | Group B |  | Group A | Group B |
| 0.0 |  | 9.8% | 10.1% |  | 0.0% | 0.0% |  | 30.0% | 28.9% |  | 18.0% | 20.8% |  | 3.3% | 3.3% |  | 17.8% | 13.7% |  | 14.6% | 11.2% |
| 0.5 |  | 10.8% | 10.3% |  | 0.0% | 0.0% |  | 29.4% | 28.7% |  | 18.6% | 21.1% |  | 3.3% | 3.1% |  | 18.5% | 14.9% |  | 14.8% | 11.6% |
| 1.0 |  | 11.6% | 10.9% |  | 0.0% | 0.0% |  | 29.4% | 28.0% |  | 19.3% | 21.3% |  | 3.0% | 3.5% |  | 18.6% | 15.6% |  | 14.9% | 12.1% |
| 1.5 |  | 11.9% | 11.5% |  | 2.0% | 2.2% |  | 29.1% | 27.5% |  | 18.6% | 21.1% |  | 2.7% | 3.1% |  | 18.5% | 15.7% |  | 15.3% | 12.8% |
| 2.0 |  | 12.7% | 11.3% |  | 4.0% | 5.0% |  | 28.5% | 26.9% |  | 17.6% | 19.9% |  | 2.4% | 3.0% |  | 18.6% | 14.2% |  | 16.0% | 13.9% |
| 2.5 |  | 12.4% | 11.1% |  | 8.7% | 10.2% |  | 27.9% | 25.8% |  | 17.2% | 17.3% |  | 2.6% | 2.9% |  | 17.3% | 13.5% |  | 16.9% | 13.1% |
| 3.0 |  | 12.2% | 10.7% |  | 12.9% | 13.6% |  | 27.3% | 25.5% |  | 16.3% | 15.3% |  | 2.3% | 2.2% |  | 12.9% | 10.1% |  | 17.3% | 13.7% |
