# Supplementary material for: Effect of Behavior Modification on Outcome in Early- to Moderate-Stage Chronic Kidney Disease: A Cluster-Randomized Trial
Source: PLoS One. 2016 Mar 21;11(3):e0151422. doi: 10.1371/journal.pone.0151422 (PMC4801411; doi:10.1371/journal.pone.0151422)
Supplement: S4 Table — (DOCX) [file pone.0151422.s007.docx]

| Table S4 |  | Deatail of anti-hypertensives changes during study period | | | | | | |  |  |  |  |  |  |  |  |  |  |  |  |  |
| --- | --- | --- | --- | --- | --- | --- | --- | --- | --- | --- | --- | --- | --- | --- | --- | --- | --- | --- | --- | --- | --- |
|  |  |  |  |  |  |  |  |  |  |  |  |  |  |  |  |  |  |  |  |  |  |
| Study period | | ARB | |  | ACEI | |  | Ca antagonist | |  | beta blocker | |  | alpha blocker | |  | Methyldopa | |  | diuretics | |
| years |  | Group A | Group B |  | Group A | Group B |  | Group A | Group B |  | Group A | Group B |  | Group A | Group B |  | Group A | Group B |  | Group A | Group B |
| 0.0 |  | 61.0% | 61.3% |  | 14.0% | 14.0% |  | 64.6% | 62.5% |  | 6.8% | 8.8% |  | 6.7% | 6.5% |  | 0.7% | 0.5% |  | 12.2% | 12.2% |
| 0.5 |  | 65.3% | 65.0% |  | 15.1% | 15.4% |  | 69.7% | 67.1% |  | 7.5% | 8.5% |  | 8.5% | 7.5% |  | 0.8% | 0.8% |  | 16.1% | 16.2% |
| 1.0 |  | 69.6% | 67.2% |  | 16.0% | 14.7% |  | 70.7% | 66.1% |  | 7.4% | 8.2% |  | 9.0% | 8.1% |  | 0.8% | 1.0% |  | 17.9% | 18.4% |
| 1.5 |  | 69.6% | 69.1% |  | 16.2% | 16.0% |  | 71.7% | 68.4% |  | 7.8% | 8.4% |  | 8.6% | 8.2% |  | 1.0% | 1.1% |  | 20.6% | 21.3% |
| 2.0 |  | 67.9% | 67.0% |  | 15.8% | 15.8% |  | 70.8% | 68.9% |  | 7.8% | 8.3% |  | 7.8% | 8.5% |  | 1.0% | 1.1% |  | 19.4% | 19.7% |
| 2.5 |  | 61.8% | 62.6% |  | 15.7% | 16.4% |  | 68.2% | 64.4% |  | 7.7% | 8.3% |  | 8.7% | 7.9% |  | 0.9% | 1.1% |  | 18.9% | 19.0% |
| 3.0 |  | 62.3% | 61.1% |  | 15.9% | 16.0% |  | 66.7% | 61.7% |  | 8.3% | 8.6% |  | 8.4% | 6.2% |  | 0.5% | 0.9% |  | 17.9% | 17.3% |
